# Interaction of formin FH2 with skeletal muscle actin. EPR and DSC studies

**DOI:** 10.1007/s00249-013-0922-0

**Published:** 2013-08-15

**Authors:** Tünde Kupi, Pál Gróf, Miklós Nyitrai, József Belágyi

**Affiliations:** 1 Department of Biophysics, Medical School, University of Pécs, Szigeti str. 12, Pécs, 7624 Hungary; 2 Department of Biophysics and Radiation Biology, Semmelweis University of Medicine, IX. Tűzoltó u. 37-47, Budapest, 1095 Hungary; 3 Szentágothai Research Center, Ifjúság u. 34, Pécs, 7624 Hungary; 4 Office for Subsidized Research Units, Hungarian Academy of Sciences, Nádor u. 7, Budapest, 1051 Hungary

**Keywords:** Cytoskeleton, Formin, Actin, Maleimido-TEMPO, Electron paramagnetic resonance, Protein conformation

## Abstract

Formins are highly conserved proteins that are essential in the formation and regulation of the actin cytoskeleton. The formin homology 2 (FH2) domain is responsible for actin binding and acts as an important nucleating factor in eukaryotic cells. In this work EPR and DSC were used to investigate the properties of the mDia1-FH2 formin fragment and its interaction with actin. MDia1-FH2 was labeled with a maleimide spin probe (MSL). EPR results suggested that the MSL was attached to a single SH group in the FH2. In DSC and temperature-dependent EPR experiments we observed that mDia1-FH2 has a flexible structure and observed a major temperature-induced conformational change at 41 °C. The results also confirmed the previous observation obtained by fluorescence methods that formin binding can destabilize the structure of actin filaments. In the EPR experiments the intermolecular connection between the monomers of formin dimers proved to be flexible. Considering the complex molecular mechanisms underlying the cellular roles of formins this internal flexibility of the dimers is probably important for manifestation of their biological functions.

**Electronic supplementary material:**

The online version of this article (doi:10.1007/s00249-013-0922-0) contains supplementary material, which is available to authorized users.

## Introduction

The dynamics of the actin cytoskeleton––its rapid assembly and disassembly, which is essential for many cellular functions––is regulated in vivo by a variety of actin-binding proteins (ABPs) (Hild et al. 2010; Lappalainen 2007; Pantaloni et al. 2001; Pollard et al. 2000; Pollard and Borisy 2003). One of the actin nucleators is formin, which acts on actin and can accelerate its assembly from pools of actin monomers. Experiments using fluorescence spectroscopic and paramagnetic resonance techniques have shown that formin binds to the barbed end of actin filaments and induces a change of their flexibility (Bugyi et al. 2006; Kupi et al. 2009; Papp et al. 2006). The binding of formins to the sides of the actin filaments is less tight and stabilizes the structure of the filaments, probably by connecting neighboring protomers (Bugyi et al. 2006). The DSC transients also revealed the destabilization of actin filaments by formin (Bugyi et al. 2006). It is expected that the interaction between actin and formin is mutual in a sense that their binding affects the conformation of both proteins. Little is known about the conformational changes in formin accompanying the actin binding.

The results of spectroscopic techniques are often based on monitoring of the properties of an attached reporter molecule. For actin several labeling sites are known, among which the most frequently targeted is the Cys-374 residue of subdomain 1 (Burley et al. 1971; Thomas et al. 1979). Despite much biochemical and biophysical work on formins, spectroscopic labeling of formins has not been reported. In this work we used the FH2 domain of the mDia1 formin. This domain is responsible for the interaction between actin and formin. A paramagnetic maleimide label was attached to a cysteine residue in the FH2. We used electron paramagnetic resonance (EPR) to reveal properties of the spin-labeled formin and the consequences of its binding to actin. To characterize the corresponding global conformational changes differential scanning calorimetry (DSC) was used. The results of these measurements showed that the spin probe bound to one single SH group in mDia1-FH2. We found that formin has a flexible structure, low heat stability, and the connection between the monomers is flexible in the mDia1-FH2 dimers also. In both DSC and EPR experiments a major heat-induced conformational change of mDia1-FH2 occurred at approximately 41 °C.

## Materials and methods

### Protein preparations

The FH2 fragments of mammalian formin mDia1 were obtained by the method of Shimada et al. (2004). mDia1-FH2 is composed of 338 amino acid residues (40 kDa) (Bugyi et al. 2006). *Escherichia coli* BL21 strain was used to express mDia1-FH2 fragments. Protein expression was induced by isopropyl-β-d-thiogalactopyranoside. Further purification of mDia1-FH2 fragments were performed by size-exclusion chromatography on Sephacryl S-300. The protein concentration was determined spectrophotometrically at 280 nm; the extinction coefficient was 20.580 M ^−1^ cm^−1^ (Bugyi et al. 2006). The purified mDia1-FH2 fragments were stored at −80 °C in storing buffer (50 mM NaCl, 5 mM DTT, 5 % glycerol, 50 mM Tris–HCl, pH 7.6).

Acetone-dried powder of rabbit skeletal muscle actin was isolated from the domestic white rabbit back muscle (Spudich and Watt 1971). The actin was stored in 0.2 mM ATP, 0.1 mM CaCl_2_, 4 mM Tris–HCl, pH 8.0 (buffer A). The concentration of G-actin was determined photometrically at 290 nm by use of a Shimadzu UV 2100 spectrophotometer; the absorption coefficient was 0.63 mg^−1^ ml cm^−1^ (Houk and Ue 1974). F-actin was prepared by the addition of 2 mM MgCl_2_ and 100 mM KCl to buffer A.

### Reaction of proteins with maleimide analogues

The mDia1-FH2 sample was dialyzed in DTT-free buffer (buffer T: 50 mM NaCl, 50 mM Tris–HCl, pH 7.6). The FH2 domains of formin were incubated with *N*-(1-oxyl-2,2,6,6-tetramethyl-4-piperidinyl)-maleimide (MSL) at a protein to label molar ratio of 1:2 for 24 h at 2 °C. Unreacted labels were removed by dialysis in DTT-free buffer T. The amount of bound labels was determined comparing the double integrals of the EPR spectra of the labeled samples and an MSL solution of known concentration. Double integrals of the EPR spectra are proportional to the spin label concentration if the spectra are registered under identical conditions. Removing the free spin labels from the solution ensures that the remaining spin labels bound to proteins. Knowing the concentration of the spin label in the protein samples and the initial protein concentration, the yield of the labeling can be determined. According to our measurements approximately 50 % of mDia1-FH2 was labeled with MSL.

Actin was labeled with MSL as described elsewhere (Kupi et al. 2009; Mossakowska et al. 1988). The labeling yield, defined as the ratio of the concentration of label to the concentration of actin monomers, was typically 0.7.

On the basis of many experimental observations it is commonly accepted that reaction of MSL with SH groups of different proteins is similar to reaction of *N*-ethylmaleimide (NEM). To verify that reaction of formin with the two maleimide derivatives––MSL and NEM––results in similar actin polymerization behavior, effects of NEM-reacted and MSL-reacted formin samples were compared. The FH2 domains of formin were reacted with NEM under conditions similar to those used for MSL. The unreacted NEM was removed by dialysis in storing buffer, which contained 5 mM DTT.

### Fluorescence labeling of actin

To follow actin polymerization *N*-(1-pyrene) iodoacetamide (pyrene) was attached to the Cys-374 site of actin as described elsewhere (Criddle et al. 1985). The concentration of pyrene-labeled actin was determined photometrically at 344 nm by use of the absorption coefficient 2.2 × 10^4^ M ^−1^ cm^−1^ (Kouyama and Mihashi 1981). The labeling yield was 0.8–0.9.

### Polymerization assay

Actin polymerization in the presence or absence of formin-FH2 was tested by fluorescence polymerization assays. The time-dependent pyrene assay was performed with a Perkin Elmer LS50B luminescence spectrometer. The actin concentration was 5 μM, including 5 % pyrene actin. Unlabeled or labeled formin concentrations were 1 μM. Before the polymerization measurements the bound calcium of actin was replaced with magnesium by adding 200 μM EGTA and 50 μM MgCl_2_ and incubating the samples for 5 min. Thereafter, polymerization of magnesium–actin was initiated by addition of 1 mM MgCl_2_ and 50 mM KCl in all cases.

### EPR spectroscopy

EPR spectra were taken with an ESP 300E X-band spectrometer from Bruker Biospin (Germany). Conventional spectra were recorded by using 20 mW microwave power and 100 kHz field modulation with an amplitude of 0.15 mT. The protein samples were placed in one or two capillary tubes (Mettler ME-18552 melting point tubes); each contained 10 or 15 μl solution. The concentration of MSL–formin varied between 50 and 60 μM. The sample tubes were positioned parallel to the static magnetic field in the center of the TM 110 cylindrical cavity. A small thermocouple was inserted in one of the capillary tubes, and the temperature was regulated with a diTC2007 type temperature controller. Spectra were usually recorded at 23 ± 0.2 °C. When studying the EPR spectra of formin and formin–actin complexes as a function of temperature, the temperature was varied between 0 and 60 °C with accuracy of 0.2 °C. The spectra were evaluated with WINEPR software from Bruker and with computer software developed in our laboratory. Both software products can be used for basic spectrum evaluation, for example determination of splitting constants and linewidths, spectral subtraction, and integration. In addition, the software developed in our laboratory enables determination of correlation times in the slow-motional regime, by using the relationship between maximum hyperfine splitting of nitroxide spectra and the correlation time as given by Goldman et al. (1972). We have shown previously (Kupi et al. 2009) that for the MSL spin label a plot of the 2${\text{A}}^{\prime }_{\text{zz}}$ hyperfine splitting against inverse temperature is described by a straight line if there is no significant change in the rotational correlation time or in molecular rotation. The plot of the hyperfine splitting constants against inverse temperature is an easy way of observing a possible, significant change in molecular reorientation. Thus a significant change of the hyperfine splitting constant may result in a breakpoint on its plot of temperature dependence. To find the breakpoint of two straight lines the statistical method suggested by Jones and Molitoris (1984) was used. The method enables calculation of whether the fit with two straight lines is better than that with a single line; it also gives the mean square error of the fit. This procedure does not, however, enable determination of the activation energy associated with the change in the rotational diffusion of the molecules studied. The rotational correlation time should be used to deduce this, by use of the relationship between correlation time and the change in the hyperfine splitting constant, as proposed by Goldman et al. (1972).

### DSC measurements

Thermal unfolding of formin, formin–actin complex, and actin was monitored by use of a Setaram Micro DSC-III calorimeter. All experiments were performed between 20 and 100 °C, the heating rate was always 0.3 K/min. Conventional Hastelloy batch vessels were used for DSC measurements; the average sample volume was 800 μl. The corresponding buffer solutions of formin or F-actin were used as reference samples. The sample and reference vessels were equilibrated with a precision of ±0.1 mg. There was no need for correction between sample and reference vessels. The repeated scan of the denatured sample was used as baseline reference, which was subtracted from the original DSC curve.

In analyzing our DSC experiments the two-state irreversible model (Sanchez-Ruiz 1992, 1988a, b) was used as working hypothesis, because our experimental observation showed that DSC transitions of formin and formin bound to F-actin were irreversible. In this model it is assumed that the reaction kinetics between the native and denatured states, regarded as a two-state irreversible process, can be described by first-order kinetics. Assuming a two-state transition process the Gibbs free energy change can be taken at the maximum of the heat capacity as Δ*G* = 0. The relationship between the integrated total calorimetric enthalpy and the entropy of the denaturation at the transition midpoint (*T*_m_) can be expressed by a simple relationship: Δ*S*(*T*_m_) = Δ*H*(*T*_m_)/*T*_m_. When the enthalpy and entropy have been determined Δ*G* can be calculated at a given temperature, assuming there is no net heat capacity change on denaturation (Bruylants et al. 2005). This latter assumption seems valid, because no shift in the baselines of the DSC curves was observed. DSC traces were analyzed with PeakFit 4.0 software from Jandel Scientific to derive excess heat capacity *C*_p,ex_, transition temperature *T*_m_ and calorimetric enthalpy change Δ*H*.

## Results

### Interaction of labeled formin with actin

**Fig. 1:**
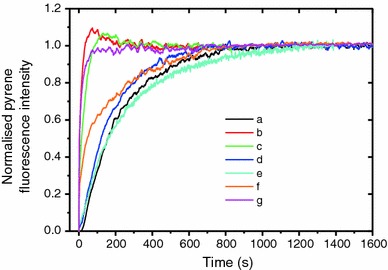
Rate of polymerization of actin by unlabelled and MSL–formin, and formin dialyzed in different buffers: (*a*) actin without formin; (*b*) actin with unlabelled formin; (*c*) actin with formin dialyzed in DTT-containing buffer T; (*d*) actin and formin dialyzed in DTT-free buffer T; (*e*) actin and MSL-labeled formin dialyzed in DTT-free buffer T; (*f*) actin and NEM-labeled formin dialyzed in DTT-containing buffer T; (*g*) actin and NEM-labeled formin not dialyzed. The protein concentrations were: 5 μM for actin and 1 μM for formin FH2 in all cases. Actin was stored in buffer A (0.2 mM ATP, 0.1 mM CaCl_2_, 4 mM Tris–HCl, pH 8.0) and labeled with pyrene (5 %). Polymerization of magnesium–actin was started by addition of 1 mM MgCl_2_ and 50 mM KCl

Experiments were performed to characterize the effect of the spin labeling of formin on its functional properties. MDia1-FH2 was labeled with MSL and polymerization of actin (5 μM, 5 % pyrene labeled) was monitored (Tobacman and Korn 1983). In the presence of unlabeled formin the rate of polymerization increased (Fig. 1). MSL-labeled formin did not significantly accelerate actin polymerization. Unlabeled formin dialyzed under DTT-free conditions was also ineffective in enhancing the rate of polymerization. NEM, an EPR-silent analogue of MSL, was also used to follow polymerization behavior. When dialysis of NEM-labeled formin was performed against DTT-containing buffer the effect of formin on actin polymerization was preserved. These observations indicated that blocking an SH group by binding of a probe did not reduce the effect of formin on the actin polymerization. Dialysis against DTT-free buffer did, however, reduce the effect of formin on the nucleation of actin.

We also tested whether the spin-labeled formin retained its actin-binding activity (Bugyi et al. 2006). MSL labeled formin was mixed with monomeric actin in a low-salt magnesium-free buffer and incubated for 6 h at 4 °C. The samples were centrifuged at 100,000×*g* and the pellets and supernatants were subjected to EPR analysis. Most (>95 %) of the labeled formin appeared in the pellets. Because formin FH2 cannot be sedimented by the applied centrifugal force, its appearance in the pellets indicated the formin was bound to actin filaments. These results also proved that the labeled formin could initiate actin polymerization. We concluded that, with the limitations described above, the experiments with spin-labeled formin can provide information about interactions between formin and actin.

### EPR measurements with MSL–formin

**Fig. 2:**
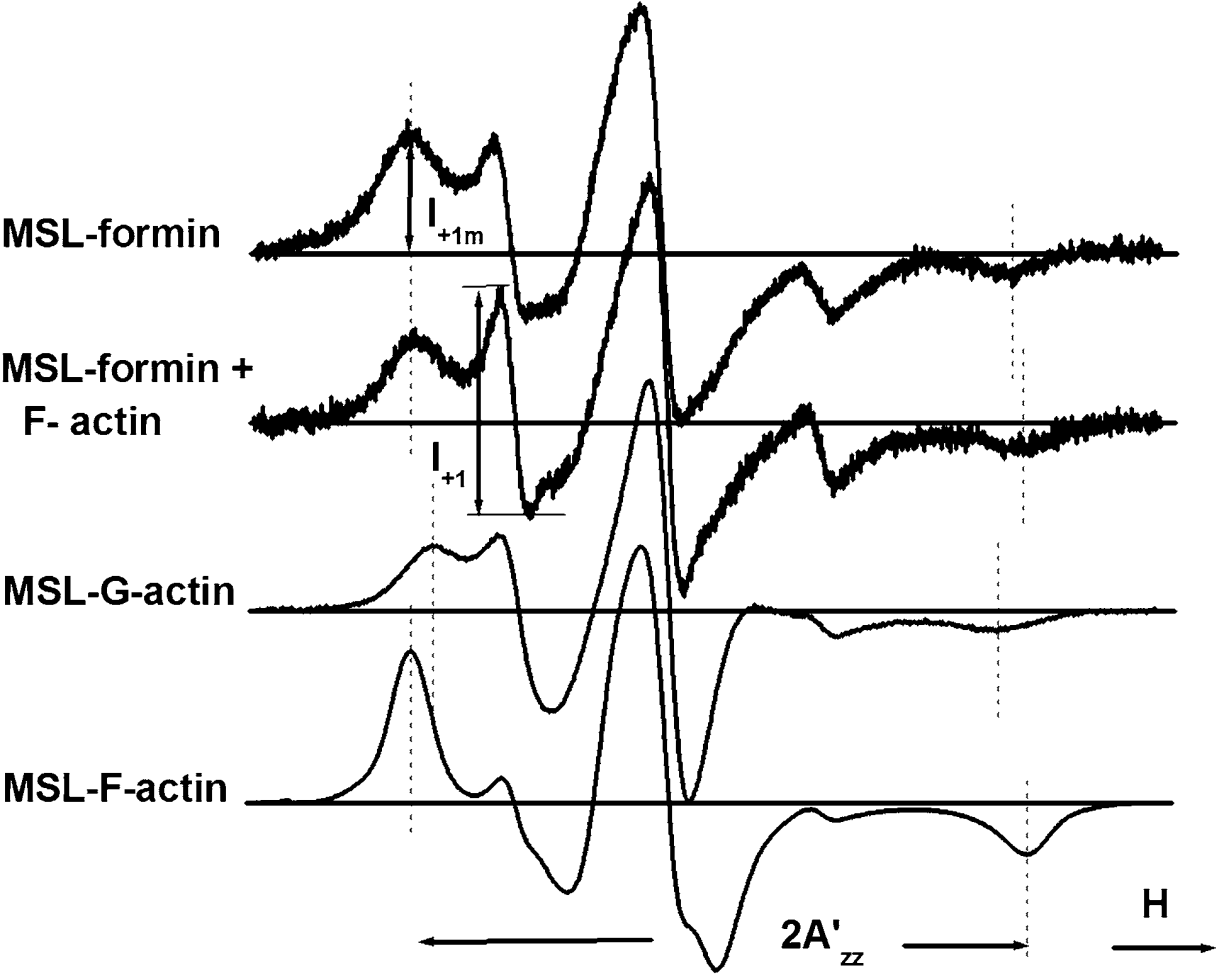
*Upper 2 spectra*: conventional EPR spectra of MSL–formin and its complex with F-actin. *Lower 2 spectra*: conventional EPR spectra of MSL–G-actin and MSL–F-actin. In contrast with MSL–F-actin, the spectra of MSL–formin contain two components with different rotational mobility. The peak heights of the low-field components are labeled *I*_+1_ and *I*_+1m_

EPR experiments were performed with MSL-labeled mDia1-FH2 formin fragments. Nitroxide maleimide reporter molecules usually react with the cysteine residues in proteins (Mossakowska et al. 1988; Thomas et al. 1975). Two EPR components were found and they were attributed to a shorter and a longer correlation time (Fig. 2). By successive subtractions of the two composite EPR spectra it was possible to estimate their relative contributions. At room temperature approximately 60 % of the signal was attributed to the component with the longer correlation time and the corresponding hyperfine splitting constant was 2${\text{A}}^{\prime }_{\text{zz}}$= 6.538 ± 0.044 mT (*n* = 4).

We calculated the rotational correlation times from the hyperfine splitting constants by separation of the outer hyperfine extreme as described elsewhere (Goldman et al. 1972). The value of the rigid limit was obtained for MSL–F-actin–formin complex in 40 % (*w*/*w*) sucrose solution at −20 °C and found to be 2${\text{A}}^{\prime }_{\text{zz}}$ = 7.104 mT. In the absence of actin the rotational correlation time for the slower component was 25.0 ns at room temperature. The other EPR component was in the fast-motional regime and was found to be 3.5 ns determined by measuring the line-heights and widths of the low and high-field lines.

### The effect of actin binding

When actin filaments were added to MSL–formin in an actin to formin molar ratio of 1:1 the value of 2${\text{A}}^{\prime }_{\text{zz}}$ increased from 6.538 mT to 6.727 ± 0.035 mT (Fig. 2). The actin-induced increase of 2${\text{A}}^{\prime }_{\text{zz}}$ of MSL–formin reflected the effect of binding of mDia1-FH2 to actin. The effect of binding did not depend significantly on the molar ratio of formin to actin between ratios of 1:5 and 1:1. Considering that under these conditions the concentration of actin filament ends is three orders of magnitude smaller (only a few nM) than the concentration of the actin protomers, most of the formins must have bound to the sides of actin filaments, and not to the filament ends. This conclusion is in agreement with observations from our sedimentation experiments and also with previous results showing that formins could bind to the sides of the actin filaments (Bugyi et al. 2006; Kupi et al. 2009).

### Temperature-dependent EPR results

**Fig. 3:**
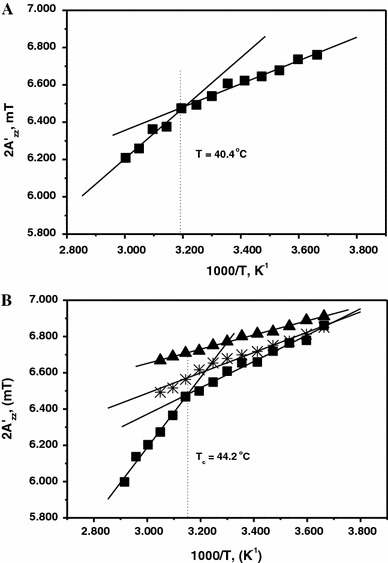
(**a**) Temperature dependence of 2${\text{A}}^{\prime }_{\text{zz}}$ of MSL–formin as a function of temperature. At approximately 41 °C a breakpoint is apparent. (**b**) Temperature dependence of the hyperfine splitting constants of: MSL–formin complex with F-actin (1:5 mol/mol) (*filled squares*), MSL–F-actin complex with formin (5:1 mol/mol) (*asterisks*), and MSL–F-actin without formin (*filled triangles*)

EPR spectra were recorded as a function of temperature between 0 and 60 °C. First, the temperature dependence of the hyperfine splitting of MSL–formin was determined in the absence of actin filaments (Fig. 3a). At 40.4 °C a breakpoint appeared in the temperature dependence of 2${\text{A}}^{\prime }_{\text{zz}}$ (Fig. 3a). Statistical analysis showed that the difference between the slopes of the two straight lines was significant by *t*-test at the *P* = 0.05 level and *F*-test declared that the linear fit with two straight lines is significantly better than a simple linear fit.

The temperature dependence of the MSL–formin spectra was then measured in the presence of actin (at 1:5 formin-to-actin molar ratio) (Fig. 3b). The hyperfine splitting constant was larger in the presence of actin at all temperatures. A breakpoint similar to that observed in the absence of actin was also found at 44.2 °C. Similar breakpoints were not observed when the actin was labeled with the spin probe either in the presence or absence of formin (Fig. 3b). Therefore, the appearance of the breakpoint reflected a major temperature-induced conformational change in formin.

### DSC measurements

**Fig. 4:**
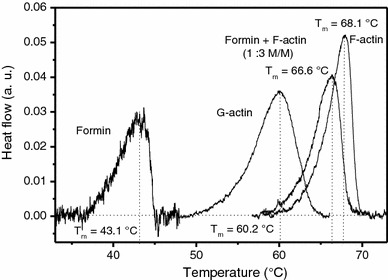
Comparison of DSC traces of formin samples: formin, G-actin, formin plus F-actin (1:3 mol/mol), and F-actin. The heat flows are plotted in arbitrary units to demonstrate the differences between the transition temperatures and the peak width at half maximum of the protein samples

To further characterize the thermodynamic properties of formin, DSC experiments were also conducted. The DSC transitions of formin and formin–actin complexes were irreversible. The DSC results for formin (96 μM) are shown in Fig. 4. The transition temperature (*T*_m_) was found to be 43.1 °C. Thermodynamic data for the transition were determined and are summarized in Table 1. The calorimetric enthalpy change (Δ*H*) was 104 kJ/mol, the entropy change (Δ*S*) was 0.33 kJ/mol K at *T*_m_, and the Gibbs free energy change (Δ*G*) was 7.7 kJ/mol at 20 °C.

**Table 1:** Thermodynamic data obtained from temperature-dependent EPR or from DSC experiments

	EPR	DSC
Δ*G* (kJ/mol)	6.7	7.7
Δ*H* (kJ/mol)	55	104
Δ*S* (kJ/mol K) at 20 °C	0.16	0.33

The table shows the Gibbs free energy changes (Δ*G*), enthalpy changes (Δ*H*), and entropy changes (Δ*S*) (the last is at 20 °C)

The transition temperature characteristic of the denaturation of actin filaments (69 μM) decreased in the presence of formin (Fig. 4). When a formin-to-actin molar ratio of 1:3 was applied the *T*_m_ value decreased from 68.1 to 66.0 °C. A similar, though smaller decrease was observed for 1:5 and 1:10 molar ratios (results not shown). These results are in agreement with previous reports that addition of formin to actin (at a formin-to-actin molar ratio of 1:20) had a destabilizing effect on actin filament structure and resulted in a shift of approximately 1.5 °C in the transition temperature (Bugyi et al. 2006).

## Discussion

### MSL probes were attached to identical labeling sites

**Fig. 5:**
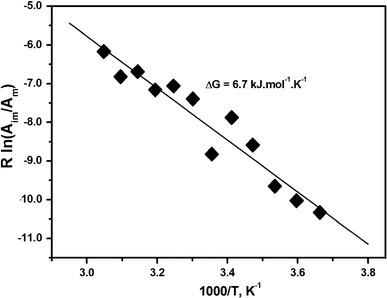
Van’t Hoff plot for the double integral ratio of the first two components in the spectrum of MSL–formin. From the slope of the straight line the free energy change (Δ*G*) was determined to be 6.7 kJ mol^−1^ K^−1^

In this work maleimide spin labels were attached to mDia1-FH2 formin fragments. Similar labeling of formin has not been reported previously. In the EPR experiments we observed two components for mDia1-FH2 (Fig. 2). To understand the origin of the two components we analyzed their contributions to the spectra obtained from temperature-dependent experiments (Fig. 3). It was possible to approximate the ratio of the double integrals of the two fractions at the m = +1 EPR transition as a function of temperature by computer manipulations (discussed in the supplementary material). The double integrals determined from the experimental spectra were temperature-independent and the natural logarithm of the ratio––double integral of the two components––showed linear dependence against reciprocal temperature (Fig. 5). This observation showed that the spin labels bound to a single cysteine residue only in formin.

To further investigate the origin of the two EPR components the corresponding rotational correlation times were calculated (3.5 and 25.0 ns). The mDia1-FH2 monomers have a molecular weight of 40 kDa and this protein adapts its functional form in dimers (Bugyi et al. 2006). The shorter correlation time (3.5 ns) was too short to assign to the entire formin and probably reflected the motion of a small segment of the protein. The longer rotational correlation time was 25 ns for MSL–formin. Actin monomer (Mw ~43 kDa) is an almost spherical protein and its corresponding correlation time is ~16–30 ns, as measured by EPR and fluorescence techniques (Belagyi and Grof 1983; Nyitrai et al. 1997). The rotational correlation time is proportional to the cube of the effective hydrodynamic radius, and thus to the molecular weight of the rotating object. For formin dimers rotating as spherical single entities one would expect values at least two times greater than for the monomers. Because formin dimers are crescent shaped and not spherical (Shimada et al. 2004) their characteristic correlation times should be even longer. On the basis of these considerations the 25.0 ns rotational correlation time was not attributed to the formin dimers, but to the individual monomers in dimers.

On the basis of these considerations we concluded that the MSL probes bound to identical residues and the two EPR components appeared because of the different rotational modes characteristic for the mDia1-FH2. From our data it is very difficult to identify the molecular events that contributed to the appearance of the two components. A possible explanation is that two distinct conformations of mDia1-FH2 co-existed in the samples. In one of the conformations the spin probe attached to a flexibly connected protein part and the rotational motion was fast. In the other co-existing conformation the protein matrix of the formin monomer was more compact and the probe reflected the wobbling rotation of the whole monomer. An alternative explanation would assume that formin existed in only one conformation in which the spin probe is located in a part of the protein that is loosely attached to the core protein fold. In this case the EPR signal reflects both the rotation of the entire monomer and that of the smaller part together, and the two components are characteristic of the same formin conformation. The conclusions we derived above from the experimental data would be the same in either of these alternative cases.

### Formin has low heat stability and a flexible structure

**Fig. 6:**
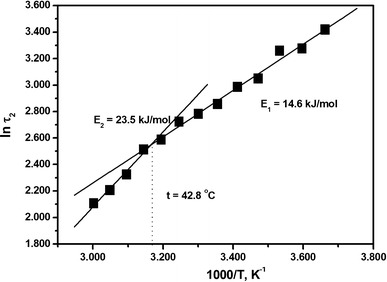
Change of the natural logarithm of the rotational correlation time for the MSL–formin complex with F-actin (1:5 mol/mol) as a function of temperature. Note the change of the activation energy after the breakpoint

**Fig. 7:**
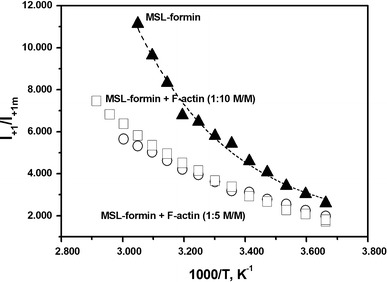
Plots of *I*_+1_/*I*_+1m_ against reciprocal absolute temperature. *Filled triangles*, MSL–formin; *squares*, MSL–formin complex with F-actin (1:10 mol/mol); *circles*, MSL–formin complex with F-actin (1:5 mol/mol)

The line shape of the EPR spectra of MSL–formin depended on temperature. At approximately 41 °C a characteristic heat-induced conformational change was detected, which indicated loosening of the formin structure (Fig. 3a). Addition of F-actin to MSL–formin increased the hyperfine splitting constant 2${\text{A}}^{\prime }_{\text{zz}}$ (Fig. 3b). The rotational correlation time (*τ*_2_) characteristic of the slower rotating component was calculated from the temperature-dependent EPR spectra obtained in the presence of actin. The logarithm of this rotational correlation time is proportional to the reciprocal of the absolute temperature (Kupi et al. 2009) and this enables calculation of the activation energy by use of an Arrhenius-type relationship. A breakpoint appeared in the function of ln*τ*_2_ vs. 1,000/T at 42.8 °C (Fig. 6). The calculated activation energies were 23.6 and 14.6 kJ/mol before and after the breakpoint, respectively (Fig. 6; Table 1). These values are connected to the rotational diffusion as Gibb’s free energies. To better understand the origin of the two subpopulations and how they behave on interaction with actin we analyzed their contributions to the spectra (discussed in the supplementary material). Changes in the relative contributions of the two subpopulations can approximately be characterized by calculating the ratio of *I*_+1_/*I*_+1m_. Here *I*_+1m_ is the peak height of the low-field maximum in the spectrum of the MSL–formin, and *I*_+1_ is the peak-to-peak height of the first component of the spectrum characterizing the faster component of the EPR spectrum (Fig. 2). The analyses revealed that actin binding tends to increase the contribution of the slower component (Fig. 7). Integrated intensities of the separated components were used to determine the relative contributions and basic thermodynamic data for MSL–formin. The corresponding thermodynamic data (Δ*H*, Δ*S*, and Δ*G*) were determined from the temperature dependence of the contribution of the two populations (Fig. 5 and supplementary material). The values are summarized in Table 1 and agree with those found in DSC experiments (Table 1). The relatively small differences can be accounted for the fact that the DSC technique provides information about the global changes in protein structure, whereas the EPR technique also reports local changes around the labeled site at different temperatures. Privalov and Potekhin (1986) and Sanchez-Ruiz (1992) assumed that the process responsible for the irreversible step has a much lower enthalpy change than the unfolding process. This assumption would explain the near agreement of the EPR results with the DSC measurements.

These experiments on temperature-dependence corroborated our previous conclusion that formin has a flexible structure.

### The connection between the formin monomers in dimers is flexible

The heat transition temperature (*T*_m_) for formin was 43.1 °C in the absence of actin (Fig. 4), similar to that indicated by the EPR data (~41 °C). In the temperature-dependent EPR measurements the differences between the 2${\text{A}}^{\prime }_{\text{zz}}$values below and above 41 °C were relatively small, which indicated that in this transition the rotational mobility of the labels changed only moderately. Thus the heat-induced conformational transition of formin had only a relatively small effect on the protein environment around the spin labels, suggesting that the protein environment was already flexible before denaturation.

The longer rotational correlation time (25 ns) was attributed to the rotation of one formin monomer, indicating that the two formin monomers could wobble almost independently from each other in the dimers. The independent motion of the two monomers assumes they do not substantially restrict each other’s motional freedom, and thus the connection between the two monomers must be flexible in the mDia1-FH2 dimers. Formin dimers can processively move with the plus ends of growing actin filaments (Moseley et al. 2004; Zigmond 2004). This behavior assumed their constant connection to the ends, but at the same time requires their ability to allow further actin monomers to bind to the plus ends of the actin filaments. To fulfill the structural criteria for these complex molecular mechanisms we believe the loose connection between the composing monomers is important and is, thus, of essential structural importance (Xu et al. 2005).

### Formin binding destabilizes the actin filaments

According to the DSC measurements formin binding reduced the transition temperature (*T*_m_) characteristic of the actin filaments (Fig. 4), indicating that the structure of the filaments became less resistant to heat denaturation. This observation is in agreement with our previous results (Bugyi et al. 2006). In the DSC experiments with formin–actin complexes the concentration of the formin was low (~10 μM) and thus the heat transition attributed to the formin denaturation was not observed. In the absence of actin we observed a major heat-induced conformational transition in formin at 41 °C (Figs. 3b, 4). We can only speculate that in the complex with actin the formin was also partially or completely heat denatured at relatively low temperatures (approx. 41 °C). Considering its destabilizing effect on actin observed in the DSC measurements one would assume that either the formin remained bound to the actin even at temperatures above its corresponding *T*_m_ value, or the formin-induced conformational changes in actin remained after the dissociation of formin.

## Conclusions

We showed that formins can be labeled on a single cysteine residue with spin probes. Spin-labeled formin can furnish information about important aspects of the intra-molecular transitions occurring in formins and in formin–actin complexes. In temperature-dependent EPR and DSC experiments we found that mDia1-FH2 has low heat stability and a flexible protein structure. The data from EPR experiments indicated that formin monomers are connected by flexible links in the dimers, which seems to be important for fulfilling their biological functions. The data also corroborated previous observations that formin binding destabilizes the actin filaments. We expect that further developments in the labeling procedure and the choice of spectroscopic probes will result in the essential improvement of these methods, which will become powerful experimental techniques promoting understanding of the molecular mechanisms underlying the functions of formins.

## Electronic supplementary material

Below is the link to the electronic supplementary material.

Supplementary material 1 (PDF 195 kb)

## Abbreviations

EPR: Electron paramagnetic resonance spectroscopy
FH2: Formin homology 2 domain
mDia1: Diaphanous-related formin-1
MSL: Maleimide spin label
Cys: Cysteine
DSC: Differential scanning calorimetry
*T*_m_: Melting temperature
F-actin: Filamentous actin polymer
G-actin: Globular actin monomer
DTT: 1,4-Dithio-d-threitol
ATP: Adenosine-5′-triphosphate
NEM: *N*-ethylmaleimide
EGTA: Ethylene glycol tetraacetic acid
