# Supplementary material for: Interaction of formin FH2 with skeletal muscle actin. EPR and DSC studies
Source: Eur Biophys J. 2013 Aug 15;42(10):757–65. doi: 10.1007/s00249-013-0922-0 (PMC3824300; doi:10.1007/s00249-013-0922-0)
Supplement: Supplementary file 1 — Supplementary material 1 (PDF 195 kb) [file 249_2013_922_MOESM1_ESM.pdf]

SUPPLEMENT

Simulations and integrals

To understand the origin of the appearance of the two subpopulations better we analyzed their contributions to the spectra. Changes in the relative contributions of the two subpopulations can be described by calculating the ratio of I_{+1} / I_{+1m}. Here I_{+1m} is the peak height of the low-field maximum in the spectrum of the MSL-formin, and I_{+1} is the peak-to-peak height of the first component of the spectrum due to the labels reflecting rotational motion of shorter correlation time.

In order to check the validity of estimating the relative contributions of the two subpopulations by calculating the I_{+1} / I_{+1m} ratios spectral simulation was performed at five representative temperatures: 0, 15, 30, 40 and 60 ^{o}C (*Table S1*). In each case two components were taken to fit the experimental spectra by non-linear least square method developed by the Freed's group with the implementation as given in (Kupi et al. 2009). In line with the experimental observations the I_{+1}/I_{+1m} ratio increases with temperature due to the increasing relative contribution of the fast component. Because the smallest and the highest ratios were found at 0° and 60 ^{o}C, respectively, we calculated at these two temperatures the rate of deviations between the values determined either using complete spectra or the separated components. Taking the values of *Table S1* we can state that the relative error of the determination is smaller than 10 %. Our conclusion was that evaluation of the I_{+1}/I_{+1m} ratios from the experimental spectra does not result in erroneous suppositions about changes in the relative contributions of the two subpopulations. On the bases of the spectral simulation we also concluded that the rotational asymmetry (R_z compared to R_x and R_y) of the slow population increases with the temperature mainly due to alteration of the R_x and R_y components; while the variation of the R_z parameter was not necessary to arrive to the best fit from 0 ^{o}C to 60 ^{o}C.

In analyzing the I_{+1} / I_{+1m} values, smaller ones indicate smaller relative contribution of the weakly immobilized subpopulation (in terminology of the above sentences this is the subpopulation which possesses shorter correlation time). Figure 7 in the paper shows the temperature dependence of the ratio of I_{+1} / I_{+1m} determined from the experimental spectra in absence or presence of actin. The function of I_{+1} / I_{+1m} against reciprocal absolute temperature

can be approximated with a near exponential function in both cases. Breakpoint did not appear in the plots. Another important finding from these measurements was that in the presence of actin the decay constant of the exponential function was significantly smaller showing that the binding of formin to actin affected the ratio of the two populations. Thus, the probe microenvironment was sensitive to the interaction of actin with formin.

The contribution of the weakly immobilized probe was smaller at all temperatures in the presence of actin than in the absence of it. The difference increased with increasing temperature. This behavior emphasizes the stabilizing effect of actin on formin and shows that this effect was more pronounced at higher temperatures. The actin binding shifted the equilibrium between the strongly and weakly immobilized states of the formin towards the former one. The component denoted as strongly immobilized corresponds to the subpopulation having longer correlation time (~ 20 - ~ 26 ns) in the context of *Table S1,* i.e. the 'slow' component. Lack of any breakpoint on the temperature dependence of I_{+1} / I_{+1m} proves that the breakpoint temperatures are the same for the strongly and weakly immobilized populations. We found in previous experiments that the observed breakpoint temperatures were not influenced by the presence of actin (Fig. 3 and 4 in the paper), which suggests that the two populations of the formin dimers bind to identical binding sites in actin filaments.

The spectra of MSL-formin and its complex with actin showed two distinct spectral components (Fig. 1). The relative fractions of the components were temperature dependent (Fig. 5). It is possible to approximate the ratio of the double integrals of the two fractions at the m = +1 EPR transition as a function of temperature by computer manipulation. A_{im} represents the double integral of the spectral component characterizing the amount of the strongly immobilized labels at the low-field maximum, whereas A_m is the double integral of the first component of the spectrum characterizing the weakly immobilized labels. The double integral of the whole spectrum was temperature independent within the limits of experimental error. The ratio of A_{im} / A_m showed an exponential dependence as a function of reciprocal absolute temperature. The populations associated with the two fractions were used to calculate an equilibrium constant K as the ratio of A_{im} / A_m. The value of R lnK shows a linear function against T^{-1} (Fig. 7).

Table S1. *Summary of the spectral simulations*

t[°C]	component	%	τ_{ave} [ns]	log(R_x)	log(R_y)	log(R_z)	I_{+1}/I_{+1m} (complete spectrum[a])	I_{+1}/I_{+1m} (component spectrum[b])
0	slow	84	26.1	6.9657	7.1539	6.5500	3.0	2.9
	fast	16	1.3	8.1152	8.1152	8.1000		
15	slow	80.4	24.9	7.0064	7.1946	6.5500	n.d.	n.d.
	fast	19.6	1.2	8.2653	8.2653	8.0508		
30	slow	77.1	23.3	7.0640	7.2522	6.5500	n.d.	n.d.
	fast	22.9	1.6	8.3990	8.3990	8.2150		
45	slow	75.6	20.9	7.1590	7.3472	6.5500	n.d.	n.d.
	fast	24.4	0.6	8.5632	8.5632	8.3487		
60	slow	70.1	20.3	7.1866	7.3748	6.5500	10.5	11.6
	fast	29.9	0.4	8.7163	8.7163	8.5018		

τ_{ave}: the average correlation time characterizing the rotation as an effective sphere; log($R_{x,y,z}$): the logarithmic values of the rotational diffusion tensor; I_{+1}/I_{+1m}: ratio as defined in the text; a.: I_{+1}/I_{+1m} values determined from each complete spectrum measuring the intensities as they were made at the experimental spectra; b.: I_{+1}/I_{+1m} values determined by measuring the corresponding intensities on the separated, simulated components.

Reference

Kupi T, Grof P, Nyitrai M, Belagyi J (2009) The uncoupling of the effects of formins on the local and global dynamics of actin filaments. Biophys J 96:2901-11.
